# Cortical thinning in male obstructive sleep apnoea patients with excessive daytime sleepiness

**DOI:** 10.3389/fneur.2023.1019457

**Published:** 2023-03-23

**Authors:** Yezhou Li, Jing Wang, Lirong Ji, Chaohong Cheng, Tong Su, Shuqing Wu, Fei Han, Daniel J. Cox, Erlei Wang, Rui Chen

**Affiliations:** ^1^School of Biological Sciences, University of Manchester, Manchester, United Kingdom; ^2^Department of Respiratory and Critical Care, The Second Affiliated Hospital of Soochow University, Suzhou, China; ^3^Department of Sleep Centre, The Second Affiliated Hospital of Soochow University, Suzhou, China; ^4^Department of Radiology, The Second Affiliated Hospital of Soochow University, Suzhou, China; ^5^Division of Psychology, Communication, and Human Neuroscience, School of Health Sciences, Faculty of Biology, Medicine and Health, University of Manchester, Manchester, United Kingdom

**Keywords:** excessive daytime sleepiness, Epworth sleepiness scale, obstructive sleep apnoea, magnetic resonance imaging, cortical thickness

## Abstract

**Background and purpose:**

Obstructive sleep apnoea is associated with excessive daytime sleepiness due to sleep fragmentation and hypoxemia, both of which can lead to abnormal brain morphology. However, the pattern of brain structural changes associated with excessive daytime sleepiness is still unclear. This study aims to investigate the effects of excessive daytime sleepiness on cortical thickness in patients with obstructive sleep apnoea.

**Materials and methods:**

61 male patients with newly diagnosed obstructive sleep apnoea were included in the present study. Polysomnography and structural MRI were performed for each participant. Subjective daytime sleepiness was assessed using the Epworth Sleepiness Scale score. Surface-based morphometric analysis was performed using Statistical Parametric Mapping 12 and Computational Anatomy 12 toolboxes to extract cortical thickness.

**Results:**

Using the median Epworth Sleepiness Scale score, patients were divided into the non-sleepiness group and the sleepiness group. The cortical thickness was markedly thinner in the sleepiness group in the left temporal, frontal, and parietal lobe and bilateral pre- and postcentral gyri (pFWE < 0.05). There was a significant negative correlation between the cortical thickness and the Epworth Sleepiness Scale score. After adjusting for age, body mass index, and obstructive sleep apnoea severity, the Epworth Sleepiness Scale score remained an independent factor affecting the cortical thickness of the left middle temporal lobe, transverse temporal and temporal pole.

**Conclusion:**

Subjective daytime sleepiness is associated with decreased cortical thickness, and the Epworth Sleepiness Scale score may be of utility as a clinical marker of brain injury in patients with obstructive sleep apnoea.

## 1. Introduction

Obstructive sleep apnoea (OSA) is a disorder characterized by obstructive apnoeas and hypopneas caused by repetitive collapse of the upper airway during sleep. Common presentations include daytime fatigue and snoring, and OSA has been shown to be associated with many other conditions including anxiety and depression, cognitive impairment, as well as raised risks of cardiovascular and cerebrovascular comorbidities (1–5). Excessive daytime sleepiness (EDS) is the most common complaint in OSA patients seeking outpatient treatment and an important criterion for the diagnosis and treatment of OSA. Our previous study found that 50% of OSA patients complained of subjective daytime sleepiness (6). EDS, in particular, is known to be a predisposing factor for cognitive impairment, accidents, interpersonal problems, and reduced productivity among OSA patients (4, 7–10).

Several previous voxel-based morphometric (VBM) studies have attempted to examine the relationship between EDS and brain injury in patients with OSA. Dusak compared 22 OSA patients and 20 controls and reported that OSA cases exhibited smaller hippocampal volumes compared to controls and lower hippocampal volumes were associated with higher Epworth Sleepiness Scale (ESS) scores, i.e., more severe EDS (11). Subsequently, Sforza reported a similar finding that EDS is associated with brain morphology abnormalities in the bilateral hippocampal regions in older patients with OSA (12). However, while current research on EDS-related brain changes mainly focuses on the structural alterations in the hippocampal region, few studies have explored such changes across the entire brain. Given the generalized EDS-related cognitive impairments in aspects beyond memory, such as attention, concentration, and temporal orientation (13, 14), it is possible that these impairments are indicative of cortical changes elsewhere in the brain, not limited to the hippocampus. Indeed, in a recent large cross-sectional study of healthy middle-aged and elderly people, it was demonstrated that the presence of EDS was associated with both global and regional atrophy, and cortical thinning predicted by EDS was maximal in the temporal region (15). Another study that focused on gray matter volume in the ventromedial prefrontal cortex also identified a cluster negatively correlated with sleepiness (16). As such, this study sets out to examine the brain-wide EDS-related structural changes in an OSA cohort who are particularly prone to daytime sleepiness.

Methodologically, in studies of the OSA patient cohorts, surface-based morphometry (SBM) has been increasingly adopted as an alternative to the VBM approach (17, 18), as the former was designed to better captures fine changes in regional structures (19, 20). Furthermore, volume is a gross measure that captures both a region’s surface area and cortical thickness, each of which might separately convey distinct pathophysiological implications. Notably, a study by Baril et al. (18) presented for the first time consistent hypertrophic changes in diffuse brain regions correlated with degree of nocturnal hypoxemia in OSA patients, while no significant differences were found in the same cohort using the VBM approach.

In this study, we adopted the technique of surface-based morphometry (SBM) to reveal potential cortical thickness changes in middle-aged OSA patients and we hypothesized that EDS may exert a brain-wide effect on the cortical thickness of the OSA brain.

## 2. Methods

### 2.1. Participants

This study included 61 male patients (aged 25 to 60 years) who were newly-diagnosed and untreated individuals with OSA received between August 2020 to June 2021 at the sleep center of the Second Affiliated Hospital of Soochow University. Only male patients were included in the study, as previous large-sample studies found that the clinical phenotype of female OSA patients differs from that of male patients (21, 22). Moreover, another study found more severe OSA-related white matter tract damages in female patients than their male counterparts with similar OSA severity (23). Therefore, given OSA is a significantly male-dominant disorder [approximately 50%–200% higher prevalence in males (24–26)], the present study chose to include only male patients to reduce sample heterogeneity. The exclusion criteria were as follows: (1) participants with a history of neurological conditions (e.g., stroke, transient-ischemic attack, Alzheimer’s disease, or Parkinson’s disease), significant respiratory conditions (e.g., asthma, chronic obstructive pulmonary disease), or other medical conditions that might affect sleep or induce sleepiness (e.g., insomnia, depression, major depressive disorder, restless leg syndrome, central sleep apnoea); (2) participants who take benzodiazepines and other drugs that causes daytime sleepiness; (3) EDS caused by other diseases; (4) the effective polysomnography recording time was less than 7 h. The participants in the study gave informed consent and the study protocol was approved by the Research Ethics Committee of the Second Affiliated Hospital of Soochow University, Suzhou, China.

### 2.2. Polysomnography and subject EDS evaluation

The participants underwent overnight, supervised, laboratory-based video polysomnography using the Compumedics Grael multifunctional PSG monitoring system. Sleep staging and sleep-related respiratory analyses were scored manually by a registered technician according to the AASM scoring criteria (3). Apnea was defined as any airflow reduction greater than 90% that lasted longer than 10s. Hypopnea was defined as >3% desaturation from pre-event baseline or arousal. The AHI was defined as the sum of the number of apnea and hypopnea per hour of sleep. Other measures included total sleep time (TST), sleep efficiency, oxygen desaturation index (ODI), proportion of sleep time with SaO₂ < 90% (T90), lowest oxygen saturation (LSaO2), arousal index, and proportions of each sleep stage. Subjective EDS was evaluated using ESS (27) and was administered at the time of the clinical evaluation on the morning of the diagnostic PSG.

### 2.3. Magnetic resonance imaging and pre-processing

Magnetic resonance structural images were collected using a T1-weighted magnetization-prepared rapid-acquisition gradient echo sequence on a 3 T Siemens Prisma MRI scanner. Acquisition parameters for the T1-weighted sequence were as follows: 240 sagittal slices with a 256 × 256 in-plane resolution; repetition time was set at 2.3 s, echo time at 2.34 milliseconds, inversion time at 900 milliseconds, and flip angle at 8°. The voxel size was 1.0 × 1.0 × 1.0 mm^3^. The acquired structural MR images were pre-processed and analyzed using the Statistical Parametric Mapping toolbox (Wellcome Trust Centre for Neuroimaging, London, UK^1^) and the Computational Anatomy Toolbox 12 (28) in MATLAB (MathWorks, 2020). The T1-weighted images were subjected to bias field correction and tissue segmentation. The images were then normalized to the International Consortium for Brain Mapping template space.

### 2.4. Surface extraction and mesh construction

The spatially normalized images were further processed in a separate Computational Anatomy Toolbox 12 surface-based pipeline to produce a normalized surface mesh for each participant. Cortical thickness was estimated as the distance between the outer and inner gray matter boundary surfaces. The resultant images of cortical thickness were smoothed using an isotropic Gaussian kernel of 15 mm. Finally, the mean value for cortical thickness across 72 surface ROIs were extracted using the Desikan-Killiany DK40 atlas (29).

### 2.5. Statistical analyses

The smoothed cortical thickness images were entered into a general linear model at the group level, with two covariates adjusting for age and BMI. Two t contrasts were created: (1) non-sleepiness > sleepiness and (2) sleepiness > non-sleepiness. For each contrast, the threshold-free cluster enhancement approach was used with 5,000 permutations to generate a non-parametric cluster inference that is not dependent on an artificially chosen cluster-level threshold (30). This corrects for the family-wise error (FWE) in the multiple statistical tests in the mass-univariate analysis, mitigating for the false discovery effect. For the subsequent ROI analysis, correlational analyses and multiple regressions adjusting for multiple clinical covariates were conducted to investigate the association between ESS scores and the mean cortical thickness in the ROIs. Other than the SPM12 and CAT12 pipelines, all other statistical analyses were conducted in SPSS 25.0 software.

## 3. Results

### 3.1. Demographic, clinical, and sleep characteristics

Using a cut-off at median ESS score = 8.0 (Inter-quartile range = 4.0–12, see Supplemental Materials for a histogram), patients were divided into the non-sleepiness group (ESS < 9) and the sleepiness group (ESS ≥ 9) (See Supplementary Figure 1 for the histogram of ESS scores). Using the median ESS as a cut-off ensures a better sample size balance between groups.

Using a cut-off at median ESS score = 8.0 (Inter-quartile range = 4.0–12, see Supplemental Materials for a histogram), patients were divided into the non-sleepiness group (ESS < 9) and the sleepiness group (ESS ≥ 9) (See the Supplementary Figure 1 for the histogram of ESS scores). Using the median ESS as a cut-off ensures a better sample size balance between groups. The demographic and clinical characteristics of OSA patients were shown in Table 1. The ESS sleepiness scale scores were higher and the symptom of somnolence was more prevalent in the sleepiness group as expected. Age, education attainment, were similar between groups. Although there was no significant difference in the prevalence of hypertension between the two groups, the systolic and diastolic blood pressure after sleep in the sleepiness group was higher.

**Table 1:** Demographic and clinical characteristics of OSA patients.

	ESS ≥ 9 (*n* = 28)	ESS < 9 (*n* = 33)	*t*/*Z*/*χ*^2^	*p*
Epworth sleepiness scale	12.07 ± 3.41	4.21 ± 2.51	10.35	<0.001
Age, years	41.04 ± 9.04	40.21 ± 8.95	0.357	0.72
Education, years	16 (15, 16)	16 (15, 16)	−0.588	0.65
Body Mass Index, kg/m^2^	27.85 ± 3.16	26.02 ± 2.88	2.35	0.02
Hypertension, *n*, %	9 (0.321)	6 (0.182)	1.592	0.21
SBP before sleep, mmHg	131.88 ± 16.07	128.69 ± 18.3	0.796	0.43
SBP after sleep, mmHg	134.67 ± 14.81	125.09 ± 16.68	2.151	0.04
DBP before sleep, mmHg	87.92 ± 11.47	87.48 ± 15.05	0.414	0.68
DBP after sleep, mmHg	93.67 ± 14.21	85.30 ± 15.17	2.427	0.02
Diabetes, *n*, %	0 (0)	2 (0.061)	1.754	0.19
Daytime sleepiness, *n*, %	23 (0.821)	11 (0.393)	14.627	<0.001
Apnoea, *n*, %	24 (0.714)	19 (0.576)	5.766	0.02
Nocturia, *n*, %	18 (0.643)	15 (0.455)	2.163	0.14
Morning fatigue, *n*, %	17 (0.607)	13 (0.394)	2.755	0.09
Poor memory, *n*, %	12 (0.429)	10 (0.357)	1.035	0.31

Values are displayed as mean ± SD or median (Inter-quartile range). BMI, body mass index; ESS, Epworth Sleepiness Scale; SBP, systolic blood pressure; DBP, diastolic blood pressure.

Polysomnographic parameters were presented in Table 2. Patients with sleepiness showed higher levels of both sleep apnoea and intermittent hypoxia. Sleepiness and non-sleepiness subjects had similar total sleep time, sleep efficiency, and proportion of non-rapid eye movement 1 and 2 sleep, although the patients with sleepiness were found to have a lower proportion of non-rapid eye movement 3 sleep. The respiratory-associated arousal index was higher in the sleepiness group.

**Table 2:** Polysomnographic parameters of OSA patients.

Polysomnographic parameters	ESS ≥ 9 (*n* = 28)	ESS < 9 (*n* = 33)	*p*
TST, min	421 (384.13, 456.43)	426 (378.5, 505.75)	0.45
Sleep efficiency, %	87.75 (83.88, 93.98)	88.5 (78.35, 92.8)	0.95
NREM 1 sleep, %	12.9 (8.725, 27.225)	12.9 (6, 21.7)	0.37
NREM 2 sleep, %	55.75 (42.175, 61.475)	52.1 (42.3, 58.5)	0.23
NREM 3 sleep, %	20.35 (17.575, 22.85)	20.4 (16.2, 24.75)	0.003
REM Sleep, %	5.95 (0, 16.275)	12.7 (8.45, 19.85)	0.50
Arousal Index (Resp.), times·h^−1^	22.3 (6.1, 50.28)	9.9 (3.8, 15.5)	0.01
Arousal Index (Spont.), times·h^−1^	5 (2.38, 7.7)	5.9 (4.1, 9.95)	0.10
AHI, times·h^−1^	46.2 (27.85, 63.05)	26.1 (13.55, 43.4)	0.02
ODI, times·h^−1^	48.8 (20.9, 64.8)	22.2 (10.83, 42)	0.01
MinSaO_2_, %	69 (58.25, 83.25)	81 (74, 86)	0.004
TS90, %	17.8 (2.03, 33.08)	2.6 (0.5, 5.4)	0.001

Values were expressed as median (interquartile range in brackets) or count (percentage). TST, total sleep time; ODI, oxygen desaturation index; AHI, apnoea-hypopnea index; MinSaO₂, Minimum pulse oxygen saturation; TS90, Proportion Time with SaO₂ < 90%; Resp., respiratory; Spont., spontaneous; NREM, non rapid eye movement; REM, rapid eye movement.

### 3.2. Cortical thickness group comparison

**Figure 1:**
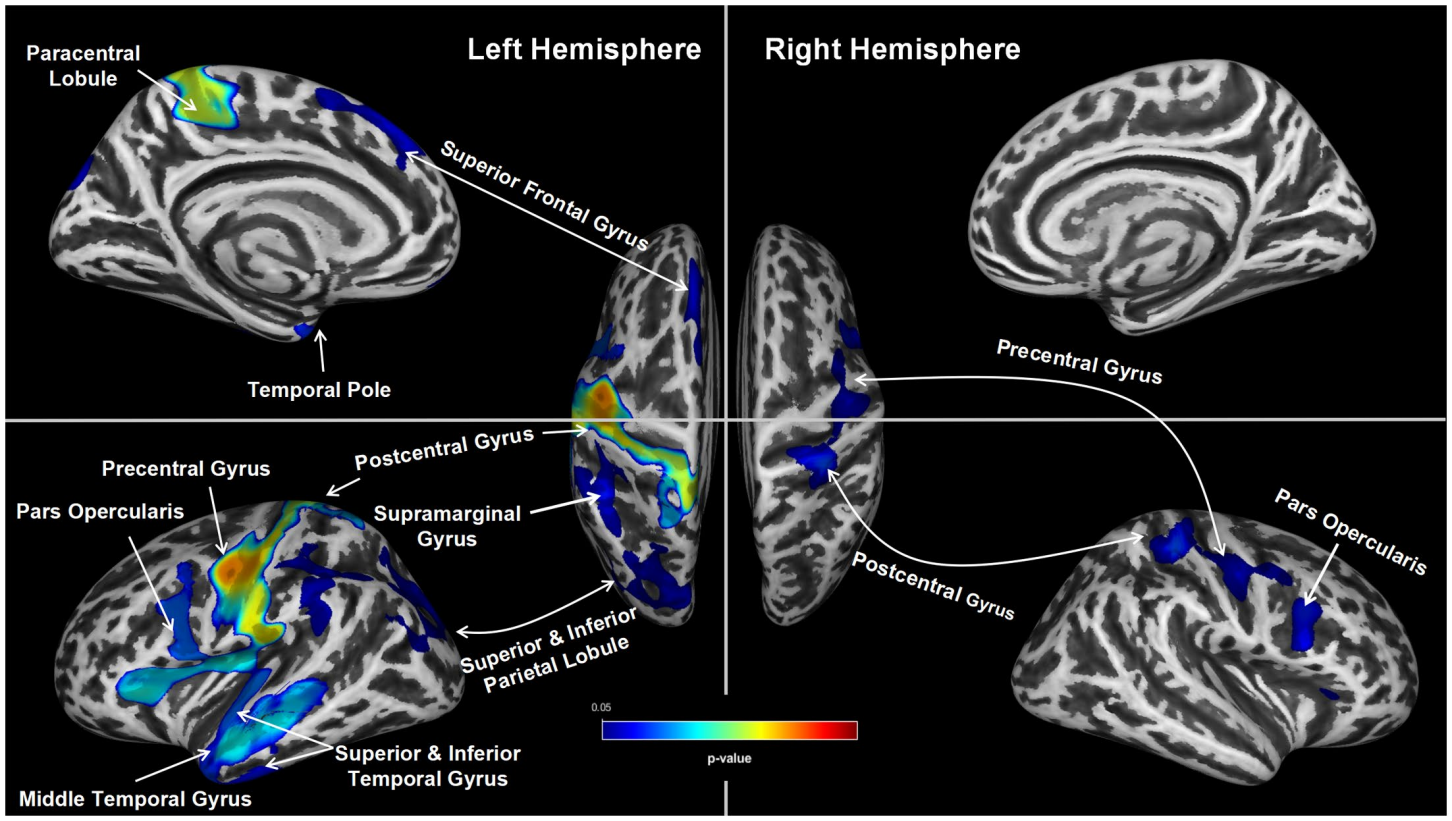
Group Differences in Cortical thickness. Group differences in average cortical thickness between the two groups (ESS ≥ 9 vs. ESS < 9). The ESS ≥ 9 group showed significantly thinner bilateral precentral gyrus, postcentral gyrus and caudal middle frontal gyrus, left superior frontal lobe, left superior, middle and inferior temporal lobe, left insula and part of the left superior and inferior parietal lobe (TFCE, pFWE < 0.05).

Findings from the statistical parametric mapping analysis are summarized in Table 3 and illustrated in Figure 1. In the non-sleepiness > sleepiness contrast, OSA patients with higher ESS score showed significant cortical thinning in bilateral precentral (left: pFWE = 0.01, right: pFWE = 0.04), postcentral (left: pFWE = 0.01, right: pFWE = 0.04) and caudal middle frontal gyrus (left: pFWE = 0.01, right: pFWE = 0.04), left superior, middle and inferior temporal lobe (pFWE = 0.01), left insula (pFWE = 0.01), left superior frontal lobe (pFWE = 0.04) and part of the left superior and inferior parietal lobe (pFWE = 0.04). For the inverse contrast of sleepiness > non-sleepiness, no significant cluster of cortical thickening was found. In addition, to cross check the results, a parallel analysis using a linear regression model was conducted and results show a similar pattern of differential brain structural changes associated with lower numeric ESS scores, albeit less statistically significant (See the Supplementary Table S1, Figure S2).

**Table 3:** Clusters of differential brain structure between groups.

Region	Cluster size	BA	pFWEc
Postcentral Gyrus (L)	5,698	1	0.01
Precentral Gyrus (L)	4,342	4	0.01
Superior Temporal Gyrus (L)	3,256	22	0.01
Supramarginal Gyrus (L)	2,862	40	0.04
Superior Parietal Lobule (L)	2,135	7	0.04
Middle Temporal Gyrus (L)	1,899	21	0.01
Superior Frontal Gyrus (L)	1,525	10	0.04
Inferior Parietal Lobule (L)	1,498	40	0.04
Insula (L)	1,357	13, 14, 16	0.01
Paracentral Lobule (L)	1,357	1	0.01
Pars Opercularis (L)	1,357	44	0.01
Inferior Temporal Gyrus (L)	1,085	20	0.01
Pars Triangularis (L)	1,085	45	0.01
Precuneus (L)	814	7	0.01
Caudal Middle Frontal Gyrus (L)	814	10	0.01
Temporal Pole (L)	543	38	0.01
Transverse Temporal Gyrus (L)	543	41, 42	0.01
Postcentral Gyrus (R)	2,162.91	1	0.04
Precentral Gyrus (R)	1,611.58	4	0.04

BA, Brodmann’s area; pFWEc, *p* values after family-wise error correction.

### 3.3. Cortical thickness and The ESS score

**Figure 2:**
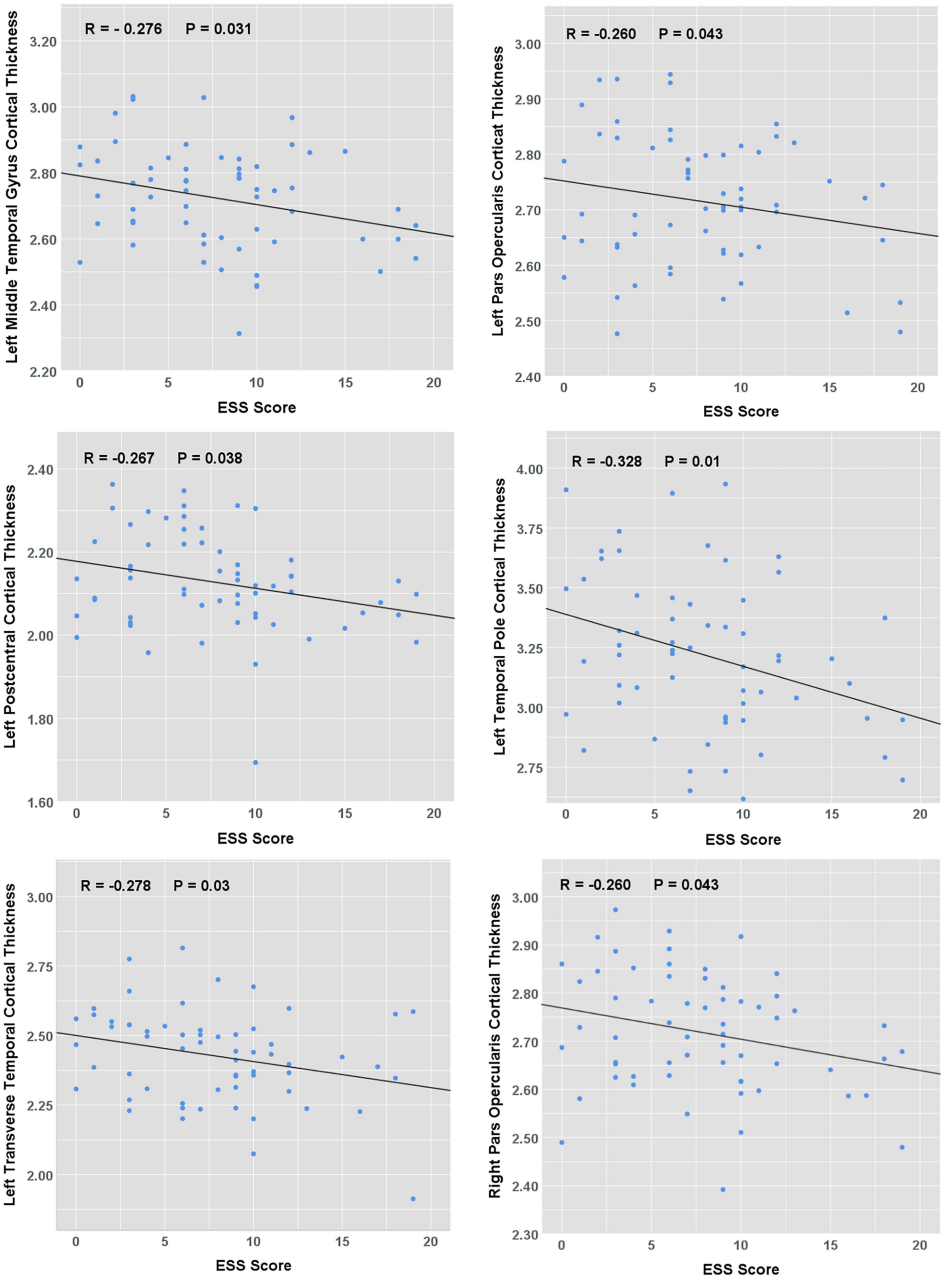
Correlation between ESS scores and cortical thickness in OSA patients. The scatterplots showing the relationship between ESS score and average cortical thickness.

A significant negative correlation between cortical thickness and ESS score was found in the left postcentral, middle temporal gyrus, temporal pole, transverse temporal, and bilateral para opercularis regions (all *p* < 0.05, shown in Figure 2). A further multiple regression analysis was conducted to adjust for the effects of age, BMI, and various polysomnography metrics of OSA severity: apnoea-hypopnea index, oxygen desaturation index, minimum pulse oxygen saturation, Proportion Time with SaO₂ < 90%, sleep efficiency and proportions of each stage of non-rapid eye movement and rapid eye movement sleep. ESS score was shown to be an independent factor affecting the cortical thickness of left middle temporal lobe (*β* = −0.292, *p* = 0.02), transverse temporal (*β* = −0.262, *p* = 0.03) and temporal pole (*β* = −0.333, *p* = 0.01).

## 4. Discussion

Excessive Daytime Sleepiness (EDS) is the cardinal symptom for which many obstructive sleep apnoea patients seek medical advice even in milder cases. According to epidemiological studies, the incidence of EDS in patients with OSA was as high as 12%–65% (7, 31–33). Our previous study also found that using a cut-off of ESS ≥ 9, approximately 50%–75% of Chinese patients with moderate to severe OSA are comorbid with EDS (6). EDS manifests in various ways, with multiple adverse behavioral health outcomes, including falling asleep while driving, a major public safety issue, and it is shown to be a contributor to increased risk of outpatient physician visits and hospitalizations (10, 34, 35). It also impacts cognitive functioning as reflected by impaired attention, alertness, memory deficit, and executive function, leading to decreased productivity, overall impaired social function, and poor quality of life (36, 37). However, no consensus has been reached on the exact mechanism of these cognitive impairments.

Our present paper is one of the earlier studies examining the association between ESS score and cortical thickness in OSA patients. We found that higher levels of ESS score were associated with decreased cortical thickness in the frontal, temporal, and parietal lobes, especially in the left hemisphere, and the results remained significant after family wise error correction based on Random Field Theory. Moreover, after adjusting for age, BMI, education, and the degree of OSA severity and hypoxia, ESS still significantly predicted the cortical thickness in the left middle and transverse temporal lobe, and the left temporal pole. That is, the degree of daytime sleepiness accounts for some additional variance in the regional cortical thickness that was not attributable to other demographic and clinical parameters. This may suggest that certain pathophysiological processes underlying EDS (see discussion below on potential mechanisms) might not be captured by the conventional polysomnography parameters that measure OSA severity and hypoxia.

These EDS-associated regional changes found in our study are in alignment with results from previous studies showing some of these regions’ involvement in cognitive impairment. For example, the cortical thinning of the superior frontal lobe has been shown to be related to dementia conversion in patients with Parkinson’s disease (38). The cortical thinning of the superior temporal gyrus was also shown to be associated with dysfunction of auditory and speech comprehension (39). In addition, parietal structural changes may be related to the inability to maintain spatial attention and visual memory impairments (40, 41). It is worth mentioning that cortical thinning was also observed in the precentral and post-central regions that control the sensation and movement of the upper airway, and structural alterations in these results could potentially contribute to the pathogenesis of OSA (42–44). Nonetheless, it should be noted that the direction of causality cannot be established from current observational evidence and further studies are needed to determine whether the sleepiness stems from the affected brain regions, or sleepiness and its underlying sleep deprivation led to the damage in these regions.

A possible mechanism that could contribute to the EDS-related brain structural changes is the reduction of slow wave sleep. Previous research has shown that patients with EDS exhibit decreased slow wave sleep, consistent with our results where the proportion of non-rapid eye movement 3 sleep was significantly lower in the sleepiness group. Slow waves in neural activity contribute to memory consolidation, and memory impairment is associated with suppressed slow waves (45, 46). During slow wave sleep, an increase in cerebrospinal fluid flow has been linked to enhanced metabolic waste removal in the brain. A reduction in slow-wave sleep would therefore lead to a decreased cerebrospinal fluid flow, the accumulation of metabolic waste, and thus brain function impairments (47). Indeed, it has been demonstrated that lower slow wave sleep duration was associated with increased generalized atrophy at autopsy (48). Moreover, studies have shown that decreased slow wave sleep is also correlated with an increase in Aβ in middle-aged and elderly healthy adults (49, 50), potentially due to the reduced metabolic waste removal. This can further increase neuronal excitability (51) and induce cortical thinning in the brain (52). Hence, a combination of the reduced cerebrospinal fluid flow and β-amyloid accumulation could offer an explanation for the cortical thinning not accounted for by any other OSA- or hypoxia-related parameters in our study.

Apart from the EDS-related structural changes, a secondary finding of the present study is that the sleepiness group suffered from more significant hypoxia, as manifested by the elevated oxygen desaturation index and reduced MinSaO2 and Proportion Time with SaO₂ < 90% (TS90%). This is consistent with our previous research results, where nocturnal hypoxemia is shown to be the main determinant of EDS in OSA patients. Many studies have shown that chronic intermittent hypoxia of OSA leads to oxidative stress, neuroinflammatory responses, damages vascular endothelial cells, affects synaptic plasticity, increases apoptosis and autophagy, and finally damages the structure and function of the central nervous system. However, not all studies so far observed hypoxic damage to the brain (18). In fact, the relationship between hypoxia and the brain may be complex. Rosenzweig et al. (53) proposed that the hypoxic processes in OSA act on the brain through a combination of adaptive and maladaptive processes, thus resulting in a mixture of gray matter hypertrophy/atrophy, and an increase/decrease in cortical thickness. For example, in processes associated with intermittent hypoxia and reactive oxygen/nitrogen species (ROS/RNS), low levels of ROS/RNS convey beneficial effects by modulatory cellular functions through factors such as HIF-1 and Nrf2 while high levels of the same species exert significant inflammatory pressure through NF-kB and AP-1 (54). In our study, we observed an unequivocal decrease in cortical thickness in OSA patients with severe EDS, suggesting that EDS may be a behavioral marker of hypoxia-induced maladaptive brain injuries, in contrast to the adaptive processes. Therefore, clinicians need to pay more attention to the changes in brain structure and functions in these OSA patients with EDS.

Lastly, methodologically, it is worth noting that this study followed the guideline laid out by a critical meta-analysis of 12 voxel-based morphometry studies in OSA patients, which attributed the lack of consistent results among studies to methodological flaws and inconsistencies (55). Specifically, in this study, high spatial resolution MR images (1 mm^3^) were used; newest available analysis software SPM12 and CAT12 with an elastic registration algorithm (Geodesic Shooting) was used; Statistical analysis was conducted with correction for multiple testing; A total of 61 participants were included in the study; covariates such as age and BMI were adjusted for in the analyses.

## 5. Limitations

This study has the following limitations. Firstly, although the present study included only male subjects to reduce sample heterogeneity given the cross-gender divergence in clinical and neuro-morphological phenotypes (see methods for details), it nevertheless comes at the cost of the comprehensiveness of the results. Secondly, an inherent shortcoming of the surface-based morphometry approach is that it captures only changes on the generalized cortical surfaces and is unable to detect changes in deep subcortical structures such as hippocampus. Future studies should employ higher resolution MR images and toolboxes that allow the examination of hippocampal subfields, to shine a light upon the subregional volumetric changes in these regions. Furthermore, our study did not include specific measures of cognitive function tests that might correlate with the observed ESS-related brain structural changes. Lastly, a more comprehensive examination in the future should also include a CPAP-treatment group to determine the potential reversibility of such brain structural alterations.

## 6. Conclusion

In conclusion, this study provides clear evidence of daytime sleepiness-associated cortical thinning in male OSA patients, with effects distributed across various subregions of the frontal, parietal and temporal lobes. In turn, this suggests ESS scores may be of clinical utility as a functional marker for early brain injuries in male OSA patients.

## Data availability statement

The raw data supporting the conclusions of this article will be made available by the authors, without undue reservation.

## Ethics statement

The studies involving human participants were reviewed and approved by Research Ethics Committee of the Second Affiliated Hospital of Soochow University, Suzhou, China. The patients/participants provided their written informed consent to participate in this study.

## Author contributions

RC and EW designed and supervised the study and revised the manuscript. JW, CC, TS, SW, and FH collected data. YL, JW, CC, and TS analyzed data. YL, JW, and DJC interpreted results and drafted the manuscript. All authors contributed to the article and approved the submitted version.

## Funding

This work was supported by the National Nature Science Foundation of China (grant numbers: 82070095, 81900085 and 81770085); The Science, Education and Health of Suzhou Youth Science and Technology Project (grant number: KJXW2021016).

## Conflict of interest

The authors declare that the research was conducted in the absence of any commercial or financial relationships that could be construed as a potential conflict of interest.

## Publisher’s note

All claims expressed in this article are solely those of the authors and do not necessarily represent those of their affiliated organizations, or those of the publisher, the editors and the reviewers. Any product that may be evaluated in this article, or claim that may be made by its manufacturer, is not guaranteed or endorsed by the publisher.

## Footnotes

^1^www.fil.ion.ucl.ac.uk/spm
