# Supplementary material for: Cortical thinning in male obstructive sleep apnoea patients with excessive daytime sleepiness
Source: Front Neurol. 2023 Mar 23;14:1019457. doi: 10.3389/fneur.2023.1019457 (PMC10076663; doi:10.3389/fneur.2023.1019457)

Supplementary Material

1.1 Supplementary Table

Linear Regression Model analysis

A parallel linear regression model was conducted using the SPM12 package and included the numeric ESS scores and age as two predictors of brain structural changes. The clusters were formed using $p<0.01$ and the cluster level p values and family-wise error corrected cluster p values were presented below in Table S1. Notably, no significant clusters were identified when (ESS^2 - ESS) was used as a predictor to check for the presence of non-linear relationships.

Table S1 Clusters of differential brain structures from the linear regression analysis for contrast -ESS

Region	Cluster Size	BA	PFWEc
Inferior Frontal Gyrus Triangularis (L)	387	45	0.899
Precentral Gyrus (L)	1236	4, 6	0.037*
Middle Temporal Gyrus (L)	1265	21, 38	0.033*
Midcingulate Cortex (R)	680	24	0.428
Postcentral Gyrus (R)	324	1	0.955
Calcarine Gyrus (L)	480	23	0.769
Entorhinal Cortex (R)	454	38	0.810
Temporal Pole (R)	351	38	0.935
Superior Orbital Gyrus (R)	270	10	0.982
Inferior Frontal Gyrus Orbitalis (R)	487	47	0.757
Inferior Frontal Gyrus Triangularis (R)	434	45	0.840
Fusiform Gyrus (L)	309	38	0.965
Rolandic Operculum (L)	226	40	0.993
Midcingulate Cortex (L)	302	24	0.969
Middle Frontal Gyrus (L)	249	46	0.989

BA, Brodmann's area; pFWEc, p values after family-wise error correction.

1.2 Supplementary Figure

Figure S1. Histogram of the ESS metric.

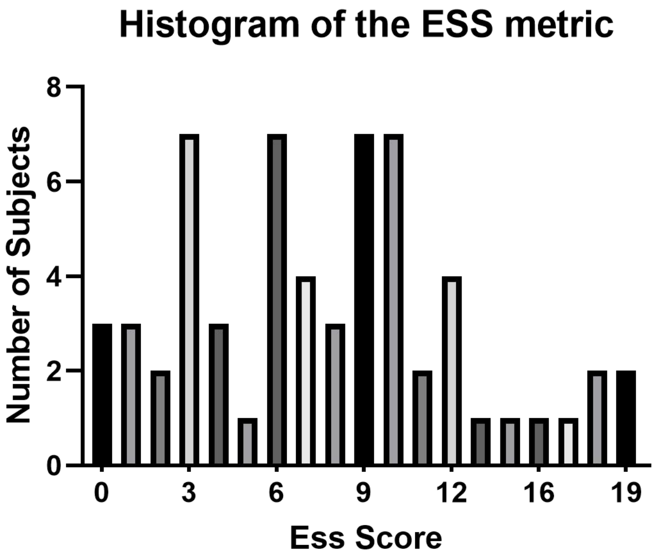

Figure S2. Regions of differential brain structures from the linear regression analysis for contrast - ESS. * pFWE<0.05

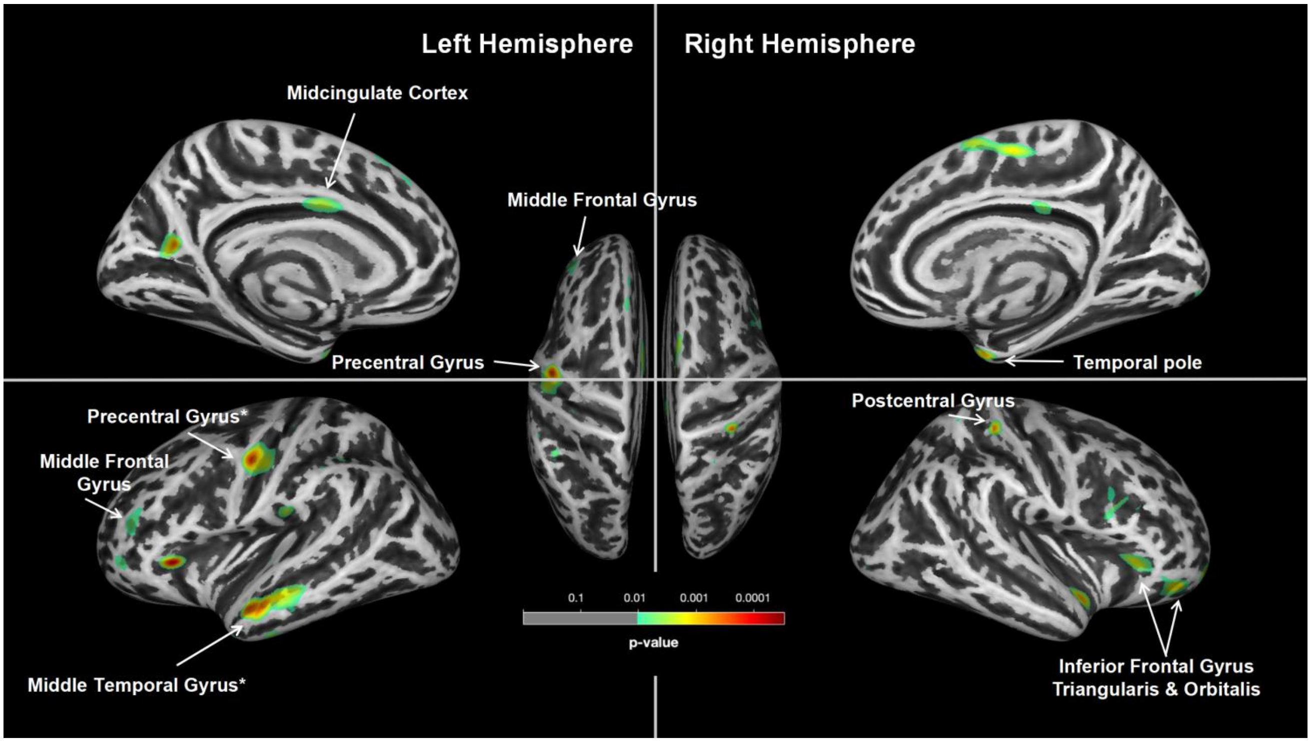

Supplement: Supplementary file 1 [file Data_Sheet_1.PDF]
